# *Panax ginseng* C. A. Meyer as a potential therapeutic agent for organ fibrosis disease

**DOI:** 10.1186/s13020-020-00400-3

**Published:** 2020-11-24

**Authors:** Hao Liu, Chongning Lv, Jincai Lu

**Affiliations:** 1 School of Traditional Chinese Materia Medica, Shenyang Pharmaceutical University, Shenyang, 110006 PR China; 2 Liaoning Provincial Key Laboratory of TCM Resources Conservation and Development, Shenyang Pharmaceutical University, Shenyang, 110006 PR China

**Keywords:** *Panax ginseng*, Liver fibrosis, Pulmonary fibrosis, Myocardial fibrosis, Renal fibrosis, TGF-β signaling pathway

## Abstract

**Background:**

Ginseng (*Panax ginseng* C. A. Meyer), a representative Chinese herbal medicine, can improve the body’s antioxidant and anti-inflammatory capacity. Recently, scientists have shifted emphasis towards the initial stages of different malignant diseases—corresponding organ fibrosis and explored the essential role of *P. ginseng* in the treatment of fibrotic diseases.

**Main body:**

In the first instance, the review generalizes the molecular mechanisms and common therapeutic methods of fibrosis. Next, due to the convenience and safety of individual medication, the research progress of ginseng extract and formulas in treating liver fibrosis, pulmonary fibrosis, myocardial fibrosis, and renal fibrosis has been systematically summarized. Finally, we describe active ingredients isolated from *P. ginseng* for their outstanding anti-fibrotic properties and further reveal the potential therapeutic prospect and limitations of *P. ginseng* in fibrotic diseases.

**Conclusions:**

*P. ginseng* can be regarded as a valuable herbal medicine against fibrous tissue proliferation. Ginseng extract, derived formulas and monomers can inhibit the abundant deposition of extracellular matrix which caused by repeated damage and provide protection for fibrotic organs. Although the molecular mechanisms such as transforming growth factor β signal transduction have been confirmed, future studies should still focus on exploring the underlying mechanisms of *P. ginseng* in treating fibrotic disease including the therapeutic targets of synergistic action of multiple components in *P. ginseng*. Moreover, it is also necessary to carry out clinical trial to evaluate the feasibility of *P. ginseng* in combination with common fibrosis drugs.

## Background

**Fig. 1:**
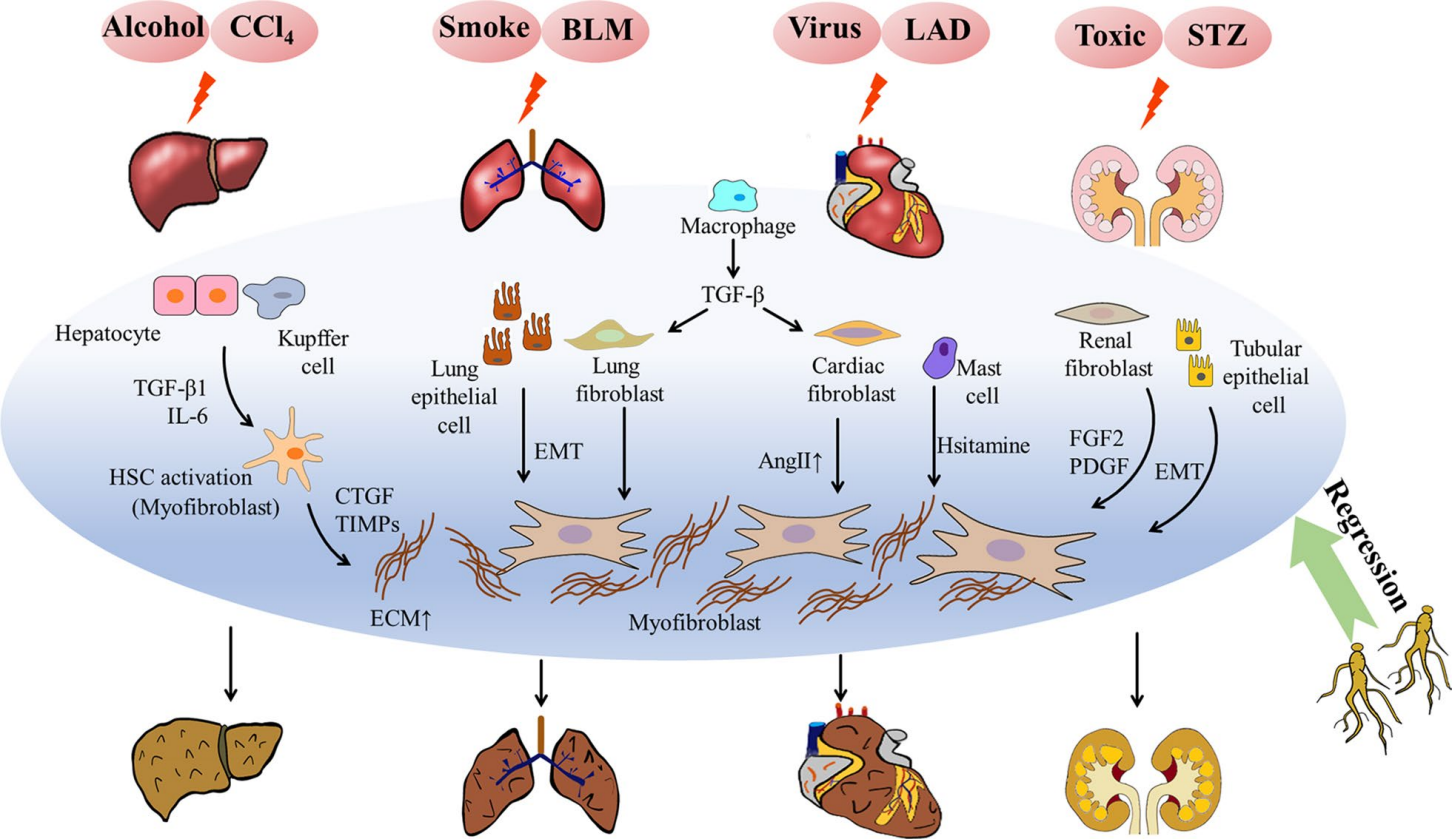
Schematic diagram showing the mechanisms and cellular events of OFD and the anti-fibrotic potential of *P. ginseng.* After receiving repeated and various stimuli, complex cellular events associated with fibrosis will occur in different organs. Activation of myofibroblasts from multiple sources will result in the abnormal deposition of extracellular matrix, indicating the initial formation of fibrosis. However, *P. ginseng* provides the possibility for the regression of fibrosis

Each organ has its own function, maintaining the normal operation of life. However, due to certain known or unknown etiologies, the appearance of organ fibrosis disease (OFD) will suddenly break this steady state. Besides the etiologies that it is hard to diagnose correctly, patients with OFD often fail to show the corresponding clinical symptoms in time, which creates difficulties for exact treatment. OFD mainly includes liver fibrosis (LF), pulmonary fibrosis (PF), myocardial fibrosis (MF), and renal fibrosis (RF), which is the initial process of serious tissue damage, organ necrosis, and major fatal diseases, dramatically shortening the lives of folks. Tissue or organ fibrosis is the leading cause of disability and death in many diseases. Statistics have shown that approximately 45% of the individuals who died of various diseases could be attributed to the results of tissue fiber hyperplasia in the developed countries [1], and even around the whole globe, organ failure caused by fibrotic diseases accounted for at least one-third of all deaths. When the tissues or organs are seriously destroyed for any unpredictable reasons, such as irritant, virus, radiation, and the damage is repetitive or exceed the self-repair capacity of cells, the entire process of OFD is initiated. Different organs have analogous fibrotic mechanism. Generally, during chronic injury, epithelial or endothelial cells secrete cytokines, for instance, tumor necrosis factor and interleukin (IL). Under this stimulation, fibroblasts will be activated and gradually transformed into myofibroblasts. After that, myofibroblasts composed of multiple sources, such as epithelial-mesenchymal transition (EMT) and resident fibroblast transformation, will abnormally proliferate and finally lead to the massive deposit of extracellular matrix (ECM), which further promote the development of fibrogenesis. Meanwhile, immune cells will produce cytokines such as transforming growth factor β1 (TGF-β)1, up-regulating the activity of myofibroblasts and boosting collagen synthesis (Fig. 1). In response to injury, the increase of fibrous connective tissue and interstitial cells and the decrease of parenchymal cells reveal the reasons of the destruction of organ structure and function [2, 3]. It may be easy to eliminate the scars appear on the skin, but in liver, lung, heart, kidney, or other internal organs, the scars cannot be easily healed. Therefore, determining suitable remedies for patients with OFD are necessary.

*Panax ginseng* C. A. Meyer is a household medicinal plant belonging to *Araliaceae*. “Panax” means all diseases can be cured in Greek, implicating the vital medicinal effect of *P. ginseng*. As an excellent preventive and therapeutic herb, *P. ginseng* has been applied medicinally for centuries in various diseases such as cancer, Alzheimer’s disease, cognitive deficit and pulmonary disease [4–7]. It can also be regarded as a tonic and functional food to improve physical function and exercise capacity or a dietary supplement to neutralize the toxic and ameliorate unexpected side effects after chemotherapy [8]. Moreover, different types of *P. ginseng* such as sun-dried ginseng, white ginseng and red ginseng exhibit different therapeutic potential.

Recently, researches have revealed the curative effect of *P. ginseng* on severe late-stage disease. However, the systematic summary of *P. ginseng* for fibrosis, the inevitable stage of disease deterioration, is still insufficient. Here, this review aims at integrating the studies of *P. ginseng* therapy for fibrosis from main organs. Notably, holistic medication can maximum exert the therapeutic potential of herbal medicine through the synergistic action of multi-component [9], so we primarily focus on ginseng extract and its derived formulas, but also consider ginseng bioactive compounds so as to further comprehensively develop the application of *P. g*inseng as an effective agent in the treatment of fibrotic diseases.

## Therapeutic strategies against OFD

Many appropriate remedies have been carried out to fibrosis intervention. Surgery can be used to remove local lesions, but not diffuse fibrosis. Also, organ transplantation is applicable for advanced fibrotic individuals. Unfortunately, it often shows high probability of organ rejection and serious complications [10]. Expelling the pathogeny is one of the most efficient approaches in treating fibrosis. After alcohol withdrawal, the clinical performance and histopathological characteristics of patient will be evidently improved, which means the control of fibrosis in liver [11].

Drug therapy has always been regarded as the dominant intervention for OFD. However, there is still no synthetic drug that can completely avoid side effects while exerting efficacy throughout the treatment process. Oral administration of pirfenidone (PFD) and nintedanib (NDB), the mainstream synthetic drugs for fibrosis, often induces hypersensitivity, nausea, fatigue, or other adverse reactions and significantly increase aspartate aminotransferase (AST) and alanine aminotransferase (ALT) in serum, implying liver function damage. PF patients may experience repeated diarrhea and gastrointestinal disorders after taking NDB, resulting in premature therapy discontinuation [12]. For hepatic fibrosis caused by schistosomiasis, praziquantel has a limitation that it often causes abdominal pain and can hardly kill immature worms at a specified dose [13]. Tenofovir and entecavir, the resultful antiviral drugs recommended by World Health Organization (WHO), act on hepatic fibrosis caused by hepatitis B virus, whereas lactic acidosis may be developed in patients with impaired liver function during therapy [14]. Calcium channel blocker, angiotensin type 1 receptor antagonist and angiotensin converting enzyme inhibitor also has unpredictable side effect in the treatment of MF [15, 16].

Most of the methods of treating fibrosis have two nonnegligible issues: toxicity and unpleasant side effects. Conversely, natural products have received attention as a better strategy for fibrosis due to its particular advantages of “green therapy” [17]. Herbal medicine was generally exploited to charm away fibrosis with satisfactory results long ago, especially in Korea, Japan, and China [18, 19]. Cheap and easily available herbs instead of synthetic drugs are used as alternative medicine in underdeveloped areas to basically guarantee fibrosis treatment. Fuzheng Huayu, a herbal medicine preparations which has finished Phase II clinical trials authorized by the U.S. Food and Drug Administration, has been proven to lighten LF by reducing α-smooth muscle actin (α-SMA) expression, activating liver natural killer cells and producing interferon-gamma [20]. Similarly, Si-Miao-Yong-An decoction (SMYAD) can restrain serine-threonine kinase Akt (AKT) and p38 mitogen-activated protein kinase (MAPK) pathway expression to dramatically reverse MF [21]. Additionally, small molecule compounds also provide vital therapeutic clues in OFD [22]. The potential of anti-inflammatory and anti-fibrosis of hesperidin derivative (HD) has been confirmed, which is based on regulating liver injury markers and depending on hedgehog-Glioma associated oncogene-1 signaling [23]. With the hepatoprotective effect, baicalin can decrease the levels of hyaluronic acid and procollagen type III in CCl_4_ model [24]. Paeoniflorin has a capacity to relieve the severity of IPF and control collagen synthesis through modulating TGF-β/Smad pathway [25]. Triptolide has an outstanding effectiveness in PF induced by paraquat through adjusting the EMT progression of epithelial cells [26]. Crocin is able to suppress the inflammation and oxidative stress in isoprenaline-induced MF by triggering toll-like receptor (TLR) 4 and nuclear factor kappa B (NF-κB) signaling [27]. Astragaloside IV or its sapogenin cycloastragenol can be a candidate ingredient of fibrosis, which is linked to the inhibition of NLRP3 inflammasome [28].

## Effects of ginseng extract and formulas on OFD

Long-term use of synthetic drug always leads to irreversible organ injury. However, more and more studies suggested that phytotherapy preserves organ function with few side effects and satisfying results. *P. ginseng*, the king of herbs, have been extensively investigated by Asian scholars. Ancient oriental healers decocted the root of *P. ginseng* with pure water as ginseng extract to cure a broad spectrum of diseases. They believed that this kind of natural and conventional method will give full play to the best nourishing effect of *P. ginseng.* Moreover, “Du Shen Tang”, a traditional decoction that uses *P. ginseng* alone and many classical formulas that combines *P. ginseng* with other adjuvant herbs were developed. So far, the compound containing various types of *P. ginseng* such as sun died, red and white ginseng obtained by the simplest preparation method has been confirmed can be available to treat OFD. Moreover, not only can ginseng extract and its formulas show a positive therapeutic effect on skin fibrosis that appears outside, but also it has a certain effect in internal organ such as liver, lung, heart and kidney [29].

### *Panax ginseng* and LF

Liver, a vital organ in the mammal body, is responsible for many critical functions such as biological metabolism, expulsion of toxins, which has been confirmed as an indispensable part of life. So losing a healthy liver signifies losing the ability of drug absorption and also losing the chance to live longer. Most of the occurrences of various liver diseases can be traced back to the continuous and uncontrolled development of LF. Numerous pathogenic factors such as hepatitis virus, alcohol, poisons and parasites can seriously damage hepatocytes and activate myofibroblasts, making the liver more susceptible to fibrogenesis and even cirrhosis. The activation of hepatic stellate cells (HSC) plays a vital key role in the mechanism of LF by irritating ECM accumulation. After the liver is stimulated by internal or external factors, TGF-β, IL and interferon-gamma will be released from hepatocytes, Kupffer cells and T cells, respectively, converting the quiescent HSC into active HSC. Thereafter, ECM with collagen in abundance begins to gather unconventionally. With the activation of HSC activity and the detrimental deposition of ECM, the fibrotic tissue has inevitably become increasingly dominant in the liver. If this condition develops out of control, it will ultimately give rise to cirrhosis and even liver cancer, endangering lives and hastening death [30].

Preliminary scientific studies have revealed that ginseng extract contributes to protect liver function from fibrosis and inhibits CCl_4_-induced liver damage in the established animal model by regulating TGF‐β1/Smad signaling pathway which involves the suppression of the TGF‐β1, Smad2, Smad3 and IL-8 expression. Meanwhile, the modification of total cholesterol and collagen expression further verified this conclusion. In line with this model, another research also investigated that chemical toxic substances could inactivate liver enzyme activities and decrease biochemical parameters, which can be reversed after taking ginseng extract [31, 32].

In addition to roots, other parts of *P. ginseng* have also been certified to confer excellent anti-fibrotic properties. Nam et al. [33] utilized a typical mild bile duct ligation model which caused liver damage by excessive producing bile acid to appraise the availability of ultrasonicated ginseng berry for LF treatment. Testing results demonstrated that the mechanisms by which ultrasonicated ginseng berry relieves LF is to inhibit the TLR4 expression and to soothe inflammation. Meanwhile, adventitious root culture of *P. ginseng* (ARCP) extracted with hot water possessed outstanding antioxidant ability after liver injury. Liver from ARCP-treated mice showed the decline of α–SMA, which reflects the mitigation of HSC transformation on the other side. AST and ALT, the popular biomarkers to assess liver injury extent, will be released from liver to serum during the formation of LF. It can be speculated that mountain ginseng adventitious root extract restrains the liver damage and LF by diminishing the levels of those markers [34].

For observing and determining the abnormal lesions in the pathological process of LF accurately, it is necessary to select appropriate staining methods. Hematoxylin-eosin staining, one of the classic means for clinicopathologic analysis of LF, obtains distinct images of cell degeneration and necrosis. Furthermore, Masson trichromatic staining can apparently distinguish blue fiber hyperplastic tissues in the histopathology and immunohistochemical staining reflects the change of HSC, indirectly implying the degree of LF. A prior study has combined these three staining methods to intuitively exhibit the pathological differences between the groups, emphasizing the anti-fibrosis and hepatoprotective ability of ginseng essence, which boiled down to attenuate oxidative stress [35].

Red ginseng, the root of fresh ginseng after steaming and drying, has been clinically given more focus as the main type of *P. ginseng* and served as a candidate medicine for hypohepatia, fatigue, myocardial ischemia and other chronic diseases. Korean scientists are devoted to the research of Korean red ginseng (KRG) and found the crude extract of KRG acts as an antioxidant and protector in Hepatitis B-induced LF [36]. Apart from internal etiologies, KRG efficaciously mitigates inflammatory responses against acute LF which caused by physical factors such as Gamma irradiation or chemical factors such as CCl_4_ and alcohol [37–39].

### *Panax ginseng* and PF

Accompanied by a high incidence, PF, also known as “chronic cancer”, is the complication of many diseases such as COVID-19. In the long course of disease, patients often die of respiratory and circulatory failure, leading to the increase of suffering and the drop of living quality. PF is a hardly reversible lung disease with multiple etiologies, for example, smoking, mineral dust, silica and harmful gas inhalation. Remarkably, previous research has summarized that smokers are much more likely to catch pulmonary fibrosis than non-smokers. Regardless of the causes, the pathological features of PF generally made up of the disorder of lung structure, the damage of normal alveolar tissue, the activation of myofibroblasts and the aberrant deposition of collagen. The whole process of PF is mainly divided into four stages, namely, clotting/coagulation stage, inflammatory cell aggregation stage, myofibroblast proliferation stage and tissue remodeling stage [40]. No matter which cytokines are secreted at any stage, it will bring about the aggregation of ECM in the subsequent stage. Leaving aside the lung transplantation, the universally used drugs such as PFD and NDB can only alleviate the development of PF rather than reverse it fundamentally. These expensive synthetic chemicals, whose side effects probably occur at uncertain times, also become a key obstacle to the remedy of PF.

Ginseng extract has been fully convinced to invigorate lung tissue and delay PF progression. By examining the changes of superoxide dismutase and malondialdehyde, Jang et al. [41] found that intragastric administration of high dose ginseng extract could resist oxidative injury in radiation-induced PF. Abundant physiological abnormal cells and necrotic cells were detected after radiation, while ginseng extract pretreatment had shown a clear decrease in the immunoreactivity of TGF-β1 and 4-hydroxynonenal in lung, exhibiting the positive impact on PF.

Owing to the excellent properties, Chinese herbal formulas with *P. ginseng* as sovereign drug are manifested to be a healing approach for PF. Renshen Pingfei Decoction contributes to the amelioration of PF in bleomycin (BLM)-damaged rats. In lung of treatment, hydroxyproline, a leading component of collagen, was progressively lower, which indirectly explained the decline of collagen synthesis. The mechanism as well as comprises interfering TGF-β1/Smad3 level [42]. Bai et al. [43] carried out an exploratory experiment to evaluate whether Jinshui Huanxian formula (JHF), an empirical recipe encompassing *P. ginseng* and other auxiliary herbs, is valuable for PF. They found gavage administration of JHF recovered lung function parameters such as forced vital capacity and lowered the levels of oxidative stress mediators by upregulating Nrf2 signaling in PF rats, which is consistent with the result of PFD. Additionly, the effect of Shenge Yangfei Capsules (SGYC) in reversing the pathophysiological changes and the deterioration of lung tissue was reported by Jia et al [44]. Compared with BLM-induced PF model, the expression of the monoamine and TGF-β1 was effectively inhibited by the positive drug dexamethasone and SGYC treatments, which are concerned with the decrease of inflammatory cytokines and ECM deposition.

Recent clinical research indicated the conventional drug used to treat PF such as glucocorticoids seemingly cannot give a satisfactory answer. Zhang et al [45]. compared a new *P. ginseng* formula which can strengthening Qi and replenishing lung (DSQRL) and prednisolone in the treatment of PF. The normalization of biochemical indicators, behavior and histological appearance provided a possibility which DSQRL may instead of glucocorticoids in PF clinical treatment. Interesting, the combined therapy of DSQRL with prednisolone involves complex drug interactions, leading the decrease of curative effect.

### *Panax ginseng* and MF

MF plays a pivotal role in cardiovascular disease which has been identified as the leading cause of death in all humanity. By reason of the lack of regenerative capacity like hepatocytes, the pivotal repair procedure of cardiomyocytes in response to cardiovascular damage is mainly manifested as MF. As the component of cardiac remodeling, MF is an abnormal pathologic change in heart, which is essentially characterized by the excess deposition of ECM between cardiomyocytes and it can also induce various heart diseases such as heart failure, sudden cardiac death and arrhythmia. The mainstream regards that there are two essential origins of MF: large-area myocardial cell death after myocardial infarction and the interstitial fibrosis. No matter which origin, with the continuous release of fibrogenic cytokines, cardiac myofibroblasts from multiple sources such as EMT and resident fibroblasts are activated by pressure overload, myocardial injury or other stimulant to proliferate in quantity [46].

Research on the heart protection of *P. ginseng* has lasted for more than half a century. Recently, Zhang et al. [47] demonstrated that ginseng crude extract represented antioxidant action to reverse the evolution of MF. The reduction of the left ventricular relative weight in ginseng group signified the breakdown of fibrotic tissue and the amelioration of MF. Moreover, ginseng treatment has no significant difference in ventricular mass indexes and oxidative stress markers with captopril, a common angiotensin converting enzyme inhibitor to treat MF. Similarly, KRG extract evidently enhanced myocardial function and relieved the severity of fibrosis in isoproterenol induced cardiac injury, which contains multiple mechanisms such as inhibited the expression of caspase-3 and TNF-α, anti-oxidative stress, reduced the water content of heart and protected cardiac cell membranes [48].

Shensong Yangxin Capsule, a prominent Chinese patent medicine rich in *P. ginseng* for treating arrhythmia, constitutes one of the contributing medicines for MF treatment. Treatment of MF with Shensong Yangxin Capsule downregulated the expression of TGF-β1, MMP-9, and collagen I and III, suppressed the transformation of fibroblasts to myofibroblasts, mitigated left atrial fibrosis and indirectly diminished the incidence of atrial fibrillation [49]. Streptozotocin (STZ) injection and ligating left anterior descending coronary artery (LAD), both of which may generate acute MF. Previous experiments proved Tongxinluo (TXL), a classical TCMF followed collateral disease theory, resulted in a significant effect in the two prevailing methods for MF model establishment. TXL can downregulate the expression of MMP-2, -9, TGF-β1 and Smad3 and upregulate the expression of Smad7 in vivo. It can also downregulate the expression of hypoxia inducible factor-1a and upregulate the expression of neuregulins-1, AKT, phosphorylation of ErbB2, ErbB4 in vitro, thereby restraining of hypoxia-induced EMT [50].

Furthermore, many studies implicated that Shengmai San/Yin provided a therapeutic strategy in atrial fibrillation, cardiac toxicity and myocardial dysfunction that caused by LAD, doxorubicin and diabetes, respectively, which result from the restrain of MF deterioration. Correlation biological mechanisms that Shengmai San/Yin exhibited its anti-fibrosis merits covers upregulating connexin expression, inhibiting PICP, PIIINP expression and TGF-β dependent pathway [51, 52]. On the basis of Shengmai San, YiQiFuMai Powder contained four additional compounds, which possesses the advanced freeze-drying technology, displayed the potential in MF treatment and ventricular remodeling. Its underlying mechanism may be linked to the intracellular signal transduction of MAPKs [53].

### *Panax ginseng* and RF

Similar to the fibrosis in other organs, RF may occur after severe kidney injury. High blood pressure, diabetes, drug poisoning and other risk factors are considered to be responsible for RF. With the development of RF, the function of kidney to excrete metabolic waste, regulate electrolyte concentration and maintain acid-base balance gradually disappears. Glomerulosclerosis and tubulointerstitial fibrosis, two principal pathological appearances of RF, have been testified to be the detrimental process leading to renal failure, including plenty of complex mechanisms. The crucial mechanism is that, during the stage of continuously renal injury, the activation of myofibroblasts from different sources will result in the abnormal amassing of ECM, accelerating the development of RF. Thereinto, in addition to interstitial fibroblasts transformation, the EMT that tubular epithelial cells transformed into myofibroblasts has also been demonstrated to be the basic source of myofibroblasts [54]. If the reversal method misses the optimal treatment period, RF will soon develop into the final phase that uremia, which will never be reversed, meaning that patients can only extend their lives slightly by dialysis.

Kalkan et al. [55] indicated that *P. ginseng* exhibited a protective action in kidney damage and fibrosis caused by broad-spectrum antibiotic. Meanwhile, ginseng extract inhibited Bax expression to regulate renal apoptosis after oral administration of gentamicin sulphate. The abatement of nephrotoxicity highlighted that treatment with *P. ginseng* may have the ability to alleviate RF caused by biological drugs. As the diet structure changes, long-term ingestion of high purine food will promote the development of kidney disease. Choi et al. [56] found sun ginseng, a species of steamed ginseng, obviously ameliorated adenine-induced renal injury by limiting the deterioration of RF, renal hypertrophy, and renal edema to a certain extent, which was in reference to the reduction of urea nitrogen and creatinine. In present findings on other types of *P. ginseng*, red ginseng was described to be suitable for both diabetes and serious comorbidity such as diabetic nephropathy caused by RF. Karunasagara et al. [57] constructed a hyperglycemia-induced diabetic nephropathy model and suggested KRG treatment modulated fibrotic and inflammatory mediators such as TGF-β1, kidney injury molecule-1, and advanced glycation end products and speeded up the process of autophagy to recover RF. Lim et al. [58] demonstrated that KRG restored e-cadherin, α-SMA and TGF-β1 expression in the treatment of RF. Oral therapy of KRG power upregulated Klotho expression in tacrolimus-induced renal injury and diminished reactive oxygen species production in mitochondrion. In addition, the red ginseng extract processed with pectin lyase also has been documented remodel renal structure and function in STZ-induced diabetic model [59].

Besides heart protective effect, TXL also been authenticated to has curative value in RF. After TXL intervention, the aberrant expression of fibronectin returned to normal in diabetic nephropathy. At the same time, the recovery pathological staining and blood indexes intuitively explained TXL can control TGF-β1 transfer from glomerular mesangial cells to glomerular endothelial cells via exosomes [60]. Additionally, the summary of experimental procedures and mechanisms of *P. ginseng* against OFD are recapitulated in Table 1 to explain for a detailed understanding.

**Table 1:** Therapeutic mechanism and anti-fibrosis actions of ginseng extract and formula

Sample	Inducing	Model	Animal groups	Therapeutic mechanisms	Organ	Ref.
Ginseng extract (GE)	CCl_4_	Wistar rat	(1) Control; (2) CCl_4_; (3) GE (400 mg/kg); 4. CCl_4_ + GE (400 mg/kg)	↓TG, TC, LDL, AST, ALT, TGF-β, TβR-I, TβR-II, Smad2, -3, -4, MMP2, -9, col 1a2, col 3a1, IL-8 ↑HDL, IL-10	Liver	31
Ginseng	CCl_4_	Wistar rat	(1) Control; (2) CCl_4_; (3) Ginseng (300 mg/kg); 4. CCl_4_ + Ginseng (300 mg/kg)	↓AST, ALT, GGT, TG, TC, Glu, caspase-3, CD68^+^ ↑TP, Calcium	Liver	32
Ultrasound ginseng berry extract (UGBE)	MBDL	SD rat	(1) Control; (2) Sham; (3) MBDL; 4. MBDL + Silymarin (150 mg/kg); 5. MBDL + GBE (250 mg/kg); 6. MBDL + UGBE (100, 250, 500 mg/kg)	↓AST, ALT, Ammonia, TNF-a, NF-κB, iNOS, NO, Myd88, TLR4 ↑SOD, GPX, CAT, HO-1	Liver	33
Tissue culture raised mountain ginseng adventitious root (TCMGAR)	CCl_4_	SD rat	(1) Control; (2) CCl_4_; 3. Cultivated ginseng (100 mg/kg); 4. TCMGAR (30, 100, 300 mg/kg)	↓AST, ALT, ALP, MDA ↑GPX	Liver	34
Ginseng essence (GE)	CCl_4_	Wistar rat	(1) Control; (2) CCl_4_; 3. CCl_4_ + Silymarin (0.5 g/kg); 4. CCl_4_ + GE (0.625, 1.25, 3.125 g/kg)	↓AST, ALT ↑GPX, GSH, GRd, GST, SOD, CAT, Albumin	Liver	35
Korean red ginseng (KRG)	γIR	C57BL/6 mouse	(1) Control; (2) γIR; 3. γIR + KRG (10, 50 mg/kg)	↓AST, ALT, GGT, ALP, COX-2, TGF-β1, ERK, NF-κB Nrf2 ↑HO-1	Liver	37
Red ginseng extract (RGE)	CCl_4_	C57BL/6 mouse	(1) Control; (2) CCl_4_; 3. CCl_4_ + RGE (30, 100, 300 mg/kg)	↓ ALT, AST, TGF-β1, PAI-1, α-SMA	Liver	38
Korean red ginseng (KRG)	Alcohol	SD rat	(1) Control; (2) Alcohol; 3. Alcohol + KRG (125, 250 mg/kg)	↓TG, TC, SREBP-1c, FAS, ACC, Leptin, HSL ↑CPT-1a, p-AMPK, PPAR-γ, Adiponectin	Liver	39
*Panax ginseng* extract (PEG)	IR	C57BL/6N mouse	(1) Control; (2) IR; 3. IR + melatonin (20 mg/kg); 4. IR + PEG (20, 50, 100 mg/kg)	↓MDA, TGF-β1, TNA-α ↑SOD, CAT, GSH	Lung	41
Renshen Pingfei Decoction	BLM	SD rat	(1) Control; (2) BLM; 3. BLM + prednisone acetate (5.4 mg/kg) 4. BLM + RPFS (0.65 g/100 g)	↓RI, Smad3, TGF-β, NF-κB, HYP, MDA ↑FVC, TLC, Cchord, SOD	Lung	42
Jinshui Huanxian formula (JHF)	BLM	SD rat	(1) Control; (2) BLM; (3) BLM + PFD (50 mg/kg) 4. BLM + JHF (10.8 g/kg)	↓α-SMA, col-I, col-III, HYP, MDA, MPO, NOX4 ↑FVC, T-SOD, GSH, CAT, Nrf2a	Lung	43
Shenge Yangfei Capsules (SGYC)	BLM	Wistar rat	(1) Control; (2) BLM; 3. BLM + dexamethasone (5 mg/kg); 4. BLM + SGYC (70, 420, 850 mg/kg)	↓MAO, TGF-β1	Lung	44
Decoction for Strengthening Qi and Replenishing Lung (DSQRL)	CCl_4_	SD rat	(1) Control; (2) CCl_4_; 3. CCl_4_ + prednisolone (6.35 mg/kg); 4. CCl_4_ + DSQRL (7.7 g/kg); 5. CCl_4_ + Prednisolone + DSQRL	↑HYP, TGF-β1, Col	lung	45
Ginseng mixture	ISO	Wistar rat	(1) Control; (2) ISO; 3. ISO + Captopril (0.45 mg/2 mL); 4. ISO + ginseng mixture (20, 80 g/kg)	↓HYP, CK, LDH, H_2_O_2_ ↑CAT, GPX	Heart	47
Korean Red Ginseng (KRG)	ISO	Wistar rat	(1) Control; (2) ISO; (3) KRG (500 mg/kg); 4. ISO + KRG (250, 500 mg/kg)	↓ HYP, LDH, AST, ALT, caspase-3, MDA, MPO, CK-MB, TNF-α ↑LVSP, SOD, CAT, GPX	Heart	48
Shensong Yangxin (SSYX)	LAD	SD rat	(1) Sham; (2) LAD; 3. LAD + SSYX (600 mg/kg)	↓col I, col III, α-SMA, TIMP-1, MMP-9, TGF-β1 ↑LVEF, LVFS	Heart	49
Tongxinluo (TXL)	LAD	SD rat	(1) Sham; (2) LAD; 3. LAD + benazepril (10 mg/kg); 4. LAD + TXL (0.2, 0.4, 0.8 g/kg)	↓ col II, col III, MMP-2, -9, α-SMA, FSP-1 ↑LVEF, LVFS, CD31, VE-cadherin	Heart	50
Shengmai San (SMS)	LAD	SD rat	(1) Sham; (2) LAD; (3) LAD + SMS (500 mg/kg)	↓col I, col III, IL-6, MCP-1, TNF-α, BNP, TGF-β1, MMP-9, α-SMA ↑LVEF, LVFS, Cx43, Cx40, TIMP-1	Heart	51
Shengmai Yin (SMY)	Doxorubicin (DOX)	SD rat	(1) Control; (2) DOX; 3. DOX + SMY (8.35, 16.7, 33.4 g/kg)	↓BNP, CK-MB, TLR2, MCP-1, INF-γ, IL-6, PICP, PIIINP ↑LVEF	Heart	52
YiQiFuMai	LAD	ICR mouse	(1) Control; (2) LAD; 3. LAD + metoprolol (5.14 mg/kg); 4. LAD + YQFM (0.13, 0.26, 0.53 g/kg)	↓MDA, LDH, CK, HYP, p38, JNK, ErK1/2, PIIINP, NT-proBNP ↑LVEF, LVFS	Heart	53
Ginseng	gentamicin sulphate (GS)	SD rat	(1) Control; (2) GS; 3. GS + KRG (100, 200 mg/kg)	↓Cre, Urea, BUN, AST, ALT, GGT, TC, TG, Glu, TP, Bax, ↑Bcl-2 Calcium, Phosphorus	Kidney	55
Sun Ginseng (SG)	adenine	SD rat	(1) Control; (2) Adenine; 3. Adenine + SG (0.5, 1.0 g/kg)	↓Scr, BUN, phosphate in serum, calcium in urine ↑calcium in serum, phosphate in urine	Kidney	56
Korean red ginseng (KRG)	STZ	SD rat	(1) Control; (2) STZ; 3. STZ + losartan (100 mg/kg/); 4. STZ + KRG (250, 500 mg/kg)	↓blood glucose, HbA1c, BUN, α-SMA, TGF-β1, KIM1, AGE, p62, mTOR ↑LC3, ATG7	Kidney	57
Korean red ginseng powder (KRGP)	tacrolimus	BALB/c mouse	(1) Control; (2) KRGP (0.2 g/kg); 3. tacrolimus; 4. tacrolimus + KRGP	↓Scr, α-SMA, TGF-β, 8-OHdG, PI3K, p-AKT, FoxO3a, ROS ↑Plasma Klotho, E-CA, MnSOD	Kidney	58
GS-E3D	STZ	SD rat	1.Control; 2. STZ; 3. STZ + GS-E3D (20, 50, 100 mg/kg)	↓albuminuria, 8-OHdG in urine, AGE in urine, α-SMA	Kidney	59
TXL	diabetes	KK-Ay mouse	1. Control: C57BL/6 mouse; 2. Model: KK-Ay mouse; 3. KK-Ay mice + TXL (0.75 g/kg)	↓TGF-β1, Smad3, col IV, FN, ↑Ccr	Kidney	60

### Effects of ginseng components on OFD

It is difficult to determine whether the efficacy of *P. ginseng* results in the synergistic interactions of multiple components or the single action of main component. However, bioactive ingredients isolated from *P. ginseng* such as saponin, polysaccharide, sesquiterpenoid and protein have been demonstrated to be a promising treatment for fibrosis (Table 2).

**Table 2:** Therapeutic mechanism and anti-fibrosis actions of ginseng components

Sample	Inducing	Model	Animal groups	Therapeutic mechanisms	Organ	Ref.
G-Rg_1_	CCl_4_, BLM, DOX, UUO	SD rat Wistar rats	(1) Control; (2) CCl_4_, BLM, TAC, UUO; 3. CCl_4_ + Rg_1_ (10, 20, 40 mg/kg); 4. BLM + prednisone acetate (5 mg/kg); BLM + Rg_1_ (18, 36, 72 mg/kg); 5. TAC + Rg_1_ (10 mg/kg); 6. UUO + Rg_1_ (12.5, 25, 50 mg/kg/d)	↓ALT, AST, LDH, ALP, HYP, α-SMA, TGF-β1, TSP-1, col I, fibronectin, p38MAPK ↑Nrf2, Cav-1, ED, FS, HIF-1, p-Akt	Liver lung heart kidney	62–65
G-Rb_1_	CCl_4_, AAC, UUO	SD rat	(1) Control; (2) CCl_4_, UUO, AAC; 3. CCl_4_ + Rb_1_ (50 mg/kg); 4. AAC + losartan (4.5 mg/kg); AAC + Rb_1_ (35, 70 mg/kg); 5. UUO + losartan (20 mg/kg); UUO + Rb_1_ (50 mg/kg)	↓AST, ALT, TG, HYP, TNF-α, IL-1β, PGE_2_, sICAM-1, p47phox, col I, fibronectin, 8-OhdG, HO-1, ANF, ACE, Ang II, AT1, TGF-β ↑IL-10	Liver heart kidney	66–68
G-Re	ISO	Wistar rat	(1) Control; (2) ISO; 3. ISO + Re (5, 20 mg/kg)	↓Smad3, col I, LVEDP, HYP ↑LVSP, +dp/dt	Heart	69
G-Rg_3_	Thioacetamide (TAA)	ICR mice	(1) Control; (2) TAA; 3. TAA + Rg_3_ (5, 10 mg/kg)	↓AST, ALT, CAT, MDA,TGF-β1, α-SMA, _P_62 ↑SOD, GSH, PI3K, AKT, mTOR	Liver	70
Ginsan	BLM	C57BL/6 mouse	(1) Control; (2) BLM; 3. BLM + Ginsan (2 mg/kg)	↓α-SMA, col-1, FN, Smad2 Smad3, TGF-β, TβRI, TβRII, ERK, Akt; ↑TβRIII	lung	72
Sesquiterpenoids from *Panax Ginseng* (SPG)	CCl_4_	ICR mouse	(1) Control; (2) CCl_4_; 3. CCl_4_ + SPG (2.5, 10 mg/kg)	↓ALT, AST, MDA, TNF-α, IL-1β, IL-6, JNK, ERK, MAPK p38, NF-κB p65, COX-2 ↑GSH, SOD, CAT	liver	74

**Fig. 2:**
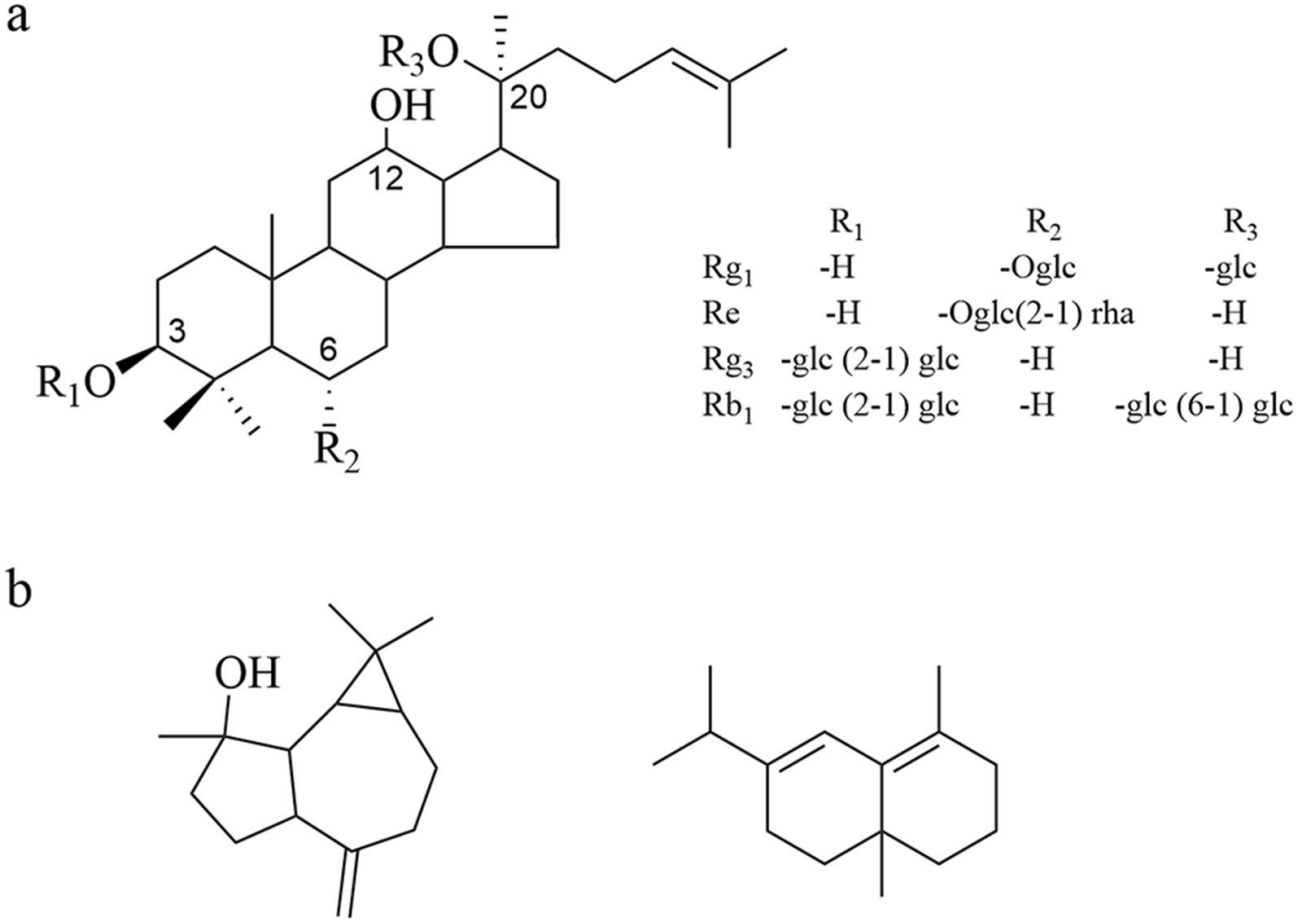
Chemical Structure of the major anti-fibrosis individual components from *P. ginseng*. **a** Ginsenoside (Rg_1_, Re, Rg_3_, Rb_1_). **b** The major sesquiterpenoids from *P. ginseng*

Currently, tremendous amounts of research illustrated the anti-fibrotic pharmacological action of *P. ginseng* is associated with ginsenosides [61]. Total ginsenoside, an extensively studied ingredient that isolated from ginseng extract with 60% ethanol following Chinese Pharmacopoeia, is deemed to be one of the popular candidates for fibrosis-associated diseases. Moreover, individual ginsenosides isolated from total ginsenoside such as Rg_1_ [62–65], Rb_1_ [66–68], Re [69], Rg_3_ [70], Mc_1_ [71] also exert their anti-fibrosis potential (Fig. 2).

**Fig. 3:**
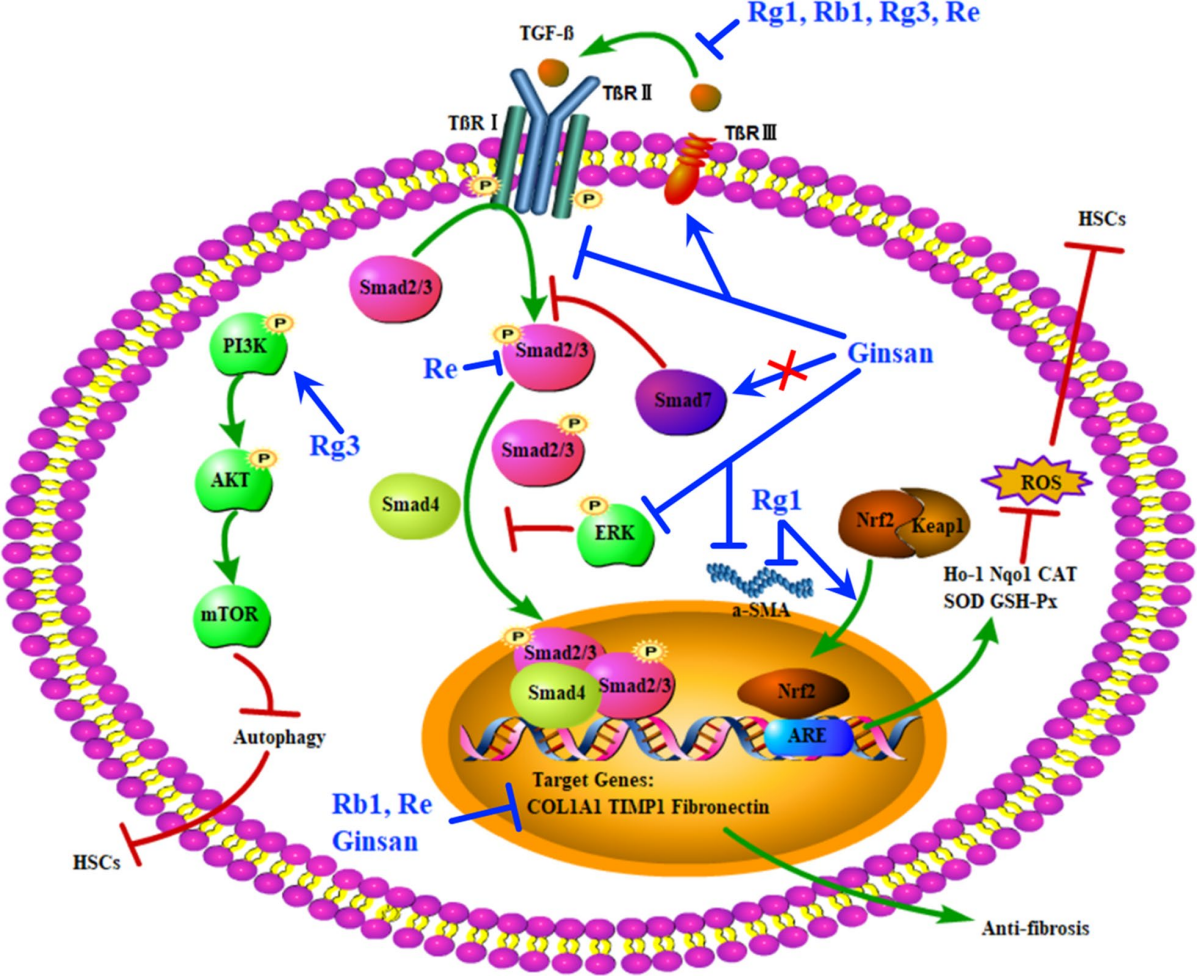
Schematic diagram summarizing the molecular mechanisms of ginseng components against fibrosis

Besides ginsenoside, ginseng polysaccharide, a biological reaction modifier, has been widely concerned in recent years. Ginsan has been corroborated to act as a therapeutic agent to alleviate symptom and establish prevention when it was used to treat Sprague–Dawley rats with PF. Whether in vivo or in vitro, the experimental results conjointly illustrated that ginsan can maintain the balance of PF-related protein expression in both TGF-β-induced and BLM-induced models [72] (Fig. 3). Ginseng protein regulated oxidative stress-induced fibroblast proliferation though restraining collagen degradation and provided protection in antifibroblast photoaging damage [73]. Sesquiterpenoids that isolated from ginseng essential oil have been described to produce hepatoprotection in CCl_4_-damaged LF. The underlying mechanisms include antioxidant and anti-inflammatory, involving NF-κB and MAPK pathways [74].

The mainstream view is that the structure of compounds contributes to their pharmacological effects, so it is necessary to determine the purification and characterization methods of the ingredients of ginseng. Individual ginsenosides, which are classified as oleanolic acid type, dammarance type and ocotillon type triterpenoid saponin, can be isolated and purified from ginseng extract by macroporous adsorptive resins and silica gel. Based on spectral and chemical analysis, the chemical structure of the ginsenosides for OFD treatment have been characterized. Their anti-fibrosis effects in different organs rely on the definable structures. For example, Rb_1_ is belong to protopanaxadiol with four sugars, while Re is belong to protopanaxatriol with three sugars. After isolated from the ethanol-insoluble fraction of ginseng extract, ginsan was purified by gel and DEAE-sephadex column chromatography, respectively. According to the hydrolysis and derivatization analysis, ginsan consists of glucose, galactose, mannose and arabinose. Meanwhile, by NMR analysis, ginsan consists of α(1→6) glucopyranoside and β(2→6) fructofuranoside. In addition, after purified by DEAE Sepharose Fast Flow, ginseng protein was further purified by Sephadex G-75 gel filtration column. It is composed of proteins with molecular weight of 27 and 13kDa which was characterized by SDS-PAGE. Sesquiterpenoids was obtained by supercritical CO_2_ extraction from *P. ginseng*. After analyzed by GC–MS, the chemical structure of the main sesquiterpenoids has been identified and shown in Fig. 2.

## Conclusion and perspective

Most of drug discovery paid more attention to the treatment of advanced disease, for instance, cirrhosis and uremia, which can hardly be cured fundamentally. But often overlooked is that, organ fibrosis is a necessary process for the development of most diseases. If it can be suppressed or reversed as much as possible, the serious condition will be alleviated or even resolved.

Many differences distinguish synthetic drugs and herbal medicines, from treatment cost to adverse reactions. Notably, only about 10% terrestrial plants have been studied in pharmacology, further indicating the prospect of herbal medicine in the discovery of anti-fibrosis [75]. With the irreplaceable medicinal value and obvious therapeutic effect, *P. ginseng* plays an increasingly significant role in the fibrotic prevention and rehabilitation and has proverbially been utilized to increase longevity. As an advantage to apply expediently, *P. ginseng* can be used independently or in combination with multiple herbs as herbal compounds, exhibiting multi-component or multi-herb combination characterizes, respectively. Whether ginseng extract or formulas, their therapeutic effect on the main organ fibrosis has been confirmed. Furthermore, saponin and polysaccharide or other monomers of ginseng exert anti-fibrosis actions by regulating fibrotic-related signal transduction pathways, which involves multiple mechanisms such as enhancing the self-healing ability of damaged cells, inhibiting fibroblast activation, promoting myofibroblast apoptosis and regulating inflammatory response. Although there may be more targets for the treatment of fibrosis in ginseng extract or formulas, a complex system of treatment, it is not as generally recognized as the ginseng monomers which have the clear structure.

It is universal believed *P. ginseng* can act as a dietary supplement or medicine to intervene disease progression. Meanwhile, synthetic drug or natural product combined with *P. ginseng* may be more advantageous than using them alone in fibrosis treatment [76]. Worth noting that drug combination has been proved to be clinical efficacy, especially in China where the integrated traditional and western medicine is widely used to treat fibrosis. Cooperation of the advantages may become one of the appropriate therapeutic methods for OFD. However, it is difficult to thoroughly determine the mechanism of drug combination, which attributes to the interaction of a wide range of complex components. In addition, *P. ginseng* can also alleviate the fibrosis caused by synthetic drugs such as omeprazole in the process of treating other diseases, although further clinical researches are needed to confirmed whether there will always be beneficial drug interactions. What matter is that patients may experience temporary stomach upset, insomnia, hypertension, or other mild adverse effects after taking an inappropriate dose of ginseng.

Overall, the preliminary experimental evidence in vivo and in vitro supported the therapeutic potential of *P. ginseng* in different organs fibrosis, which has a bearing on anti-inflammation, antioxidant and regulating TGF-β/SMAD pathway. However, in order to better apply *P. ginseng* in the clinical practice of OFD and promote the modern development of traditional Chinese medicine represented by ginseng in the treatment of fibrosis, further convincing evidence of the relationship between the all-round mechanisms and the anti-fibrotic activity of *P. ginseng* requires to be provided.

## Data Availability

Not applicable.

## Abbreviations

8-OHdG: 8-hydroxy-2′-deoxyguanosine
AAC: Abdominal aortic coarctation
ACC: Acetyl-coenzyme Acarboxylase
ACE: Ang converting enzyme
AGE: Advanced glycation end product
AKT: Serine-threonine kinase Akt
ALP: Alkaline phosphatase
ALT: Alanine aminotransferase
AMPK: AMP-activated protein kinase
AMS: Ammonium sulfate
ANF: Atrial natriuretic factor
Ang II: Angiotensin II
ARCP: Extracted adventitious root culture of P ginseng
AST: Aspartate aminotransferase
AT1: Ang II type 1
ATG7: Autophagy related 7
Bax: BCL2-Associated X Protein
Bcl-2: B-cell lymphoma-2
BLM: Bleomycin
BNP: Brain natriuretic peptide
BUN: Blood urea nitrogen
CAT: Catalase
Cchord: Chord compliance
CCl_4_: Carbon tetrachloride
Ccr: Creatinine clearance ratio
CD31: Platelet endothelial cell adhesion molecule-1
CFs: Cardiac myofibroblasts
CHM: Chinese herb medicine
CK: Creatine kinase
CK-MB: creatine kinase-MB
Col: Collagen
COX-2: Cyclooxygenase-2
CPT-1: Carnitine palmitoyl transferase-1
Cre: Creatinine
CTGF: Connective tissue growth factor
CVF: Collagen volume fraction
Cx: Connexin
E-CA: E-cadherin
ECM: Extracellular matrix
EMT: Epithelial-mesenchymal transition
ERK: Extracellular signal-regulated kinases
ET: Ethanol
FAS: Fatty acid synthase
FGF2: Basic fibroblast growth factor
FN: Fibronectin
FoxO3a: Forkhead box protein O transcription factor 3a
FSP-1: Fibroblast-specific protein 1
FVC: Forced vital capacity
FZHY: Fuzheng Huayu
GGT: Gama-glutamil transferase
Glu: Glucose
GRd: Glutathione reductase
GSH: Glutathione
GST: Glutathione S-transferase
GSX: Glutathione peroxidase
H_2_O_2_: Hydrogen peroxide
HbA1c: Glycated hemoglobin type A1c
HD: Hesperidin derivative
HDL: High-density lipoprotein
HO-1: Heme oxygenase-1
HSC: Hepatic stellate cells
HSL: Hormone sensitive lipase
HYP: Hydroxyproline
IL: Interleukin
INF-γ: Interferon gamma
iNOS: Inducible nitric oxide synthase
IPF: Idiopathic pulmonary fibrosis
IR: Irradiation
ISO: Isoproterenol
JHF: Jinshui Huanxian formula
JNK: c-Jun N-terminal kinase
KIM1: Kidney injury molecule-1
KRG: Korean red ginseng
LAD: Ligating left anterior descending coronary artery
LC3: Microtubule associated protein light chain 3
LDH: Lactate dehydrogenase
LDL: Low-density lipoprotein
LF: Liver fibrosis
LVEDP: Left ventricular end diastolic pressure
LVEF: Left ventricular ejection fraction
LVFS: Left ventricular fractional shortening
LVSP: Left ventricular systolic pressure
MAO: Monoamine
MAPK: Mitogen-activated protein kinase
MBDL: Mild bile duct ligation
MCP-1: Monocyte chemoattractant protein-1
MDA: Malondialdehyde
MF: Myocardial fibrosis
MMP: Matrix metalloproteinase
MnSOD: Manganese superoxide dismutase
MPO: Myeloperoxidase
mTOR: Mammalian target of rapamycin
Myd88: myeloid differentiation primary response gene 88
NDB: Nintedanib
NF-κB: Nuclear factor kappa B
NO: Nitric oxide
NOX4: NADPH oxid4
Nrf2: Nuclear factor erythroid 2-related factor 2
NT-proBNP: N-terminal pro-B-type natriuretic peptide
OFD: Organ fibrosis diseases
PAI-1: Plasminogen activator inhibitor 1
PDGF: Platelet-derived growth factor
PFD: Pirfenidone
PI3K: Phosphatidylinositol 3-kinase
PICP: Propeptide of procollagen type I
PIIINP: Amino terminal propeptide of procollagen type III
PPAR: Peroxisome proliferator-activated receptor
RF: Renal fibrosis
RI: Resistance
ROS: Reactive oxygen species
Scr: Serum creatinine
SGYC: Shenge Yangfei Capsules
Smad: Mothers against decapentaplegic homolog
SMYAD: Si-Miao-Yong-An
SOD: Superoxide dismutase
SQRL: Strengthening Qi and Replenishing Lung
SREBP-1c: Sterol regulatory element binding protein-1c
STZ: Streptozotocin
TAC: Transverse Aortic Constriction
TC: Total cholesterol
TCMF: Traditional Chinese medicine formula
TG: Triglycerides
TGF-β: Transforming growth factor β
TIMP: Tissue inhibitor matrix metalloproteinase
TLC: Total lung capacity
TLR: Toll-like receptor
TNF-α: Tumor necrosis factor-alpha
TP: Total protein
TXL: Tongxinluo
TβR: Transforming growth factor beta receptor
UAER: Urine albumin excretion ratio
UUO: Unilateral ureteral obstruction
WHO: World Health Organization
α-SMA: α-smooth muscle actin
